# The effect of maternal depressive symptoms on infant feeding practices in rural Ethiopia: community based birth cohort study

**DOI:** 10.1186/s13006-021-00375-3

**Published:** 2021-03-20

**Authors:** Yitbarek Kidane Woldetensay, Tefera Belachew, Shibani Ghosh, Eva Johanna Kantelhardt, Hans Konrad Biesalski, Veronika Scherbaum

**Affiliations:** 1 Institute of Nutrition Science (140a), University of Hohenheim, Stuttgart, Germany; 2 Food Security Center, University of Hohenheim, Stuttgart, Germany; 3 Department of Population and Family Health, College of Health Sciences, Jimma University, Jimma, Ethiopia; 4 Tufts University, Friedman School of Nutrition Science and Policy, Boston, USA; 5 Department of Gynecology, Faculty of Medicine, Martin-Luther University, Halle, Germany; 6 Institute of Medical Epidemiology, Biostatistics, and Informatics, Faculty of Medicine, Martin-Luther University, Halle, Germany

**Keywords:** Infant feeding practices, Prenatal depression, Postnatal depression, Household food insecurity, Intimate partner violence, Social support, Ethiopia

## Abstract

**Background:**

Maternal depression and other psychosocial factors have been shown to have adverse consequences on infant feeding practices. This study explored the longitudinal relationship of maternal depressive symptoms and other selected psychosocial factors with infant feeding practices (IFPs) in rural Ethiopia using summary IFP index.

**Methods:**

This study uses existing data from the ENGINE birth cohort study, conducted from March 2014 to March 2016 in three districts in the southwest of Ethiopia. A total of 4680 pregnant women were recruited and data were collected once during pregnancy (twice for those in the first trimester), at birth, and then every 3 months until the child was 12 months old. A standardized questionnaire was used to collect data on IFPs, maternal depressive symptoms, household food insecurity, intimate partner violence (IPV), maternal social support, active social participation, and other sociodemographic variables. A composite measure of IFP index was computed using 14 WHO recommended infant and young child feeding (IYCF) practice indicators. High IFP index indicated best practice. Prenatal and postnatal maternal depressive symptoms were assessed using the patient health questionnaire (PHQ-9). Linear multilevel mixed effects model was fitted to assess longitudinal relationship of IFPs with maternal depression and other psychosocial factors.

**Results:**

Reports of higher postnatal depressive symptoms (ß = − 1.03, *P* = 0.001) and IPV (ß = − 0.21, *P* = 0.001) were associated with lower scores on the IFP index. Whereas, reports of better maternal social support (ß = 0.11, *P* = 0.002) and active social participation (ß = 0.55, *P* < 0.001) were associated with higher scores on the IFP index. Contrary to expectations, moderate household food insecurity (ß = 0.84, *P* = 0.003), severe household food insecurity (ß = 1.03, *P* = 0.01) and infant morbidity episodes (ß = 0.63, *P* = 0.013) were associated with higher scores on the IFP index.

**Conclusions:**

Overall, a multitude of factors are related to IFPs and hence coordinated, multi-sectoral and multi-stakeholder interventions including maternal depressive symptoms screening and management are needed to improve infant feeding practices.

## Background

According to recent WHO reports, significant global progress has been made in reducing child mortality since 1990 [1]. The global under-5 mortality rate has dropped by 59% between 1990 and 2018. However, there are still disparities in under-5 mortality across regions and countries. Sub-Saharan Africa remains the region with the highest rate in the world. Half of all under-five deaths in 2018 occurred in just five countries: India, Nigeria, Pakistan, Ethiopia and the Democratic Republic of Congo. Nutrition-related factors contribute to about 45% of deaths in children under-5 years of age [1].

Nutritional deficits during the first 2 years of life are associated with stunting, leading to the adult being shorter than his or her potential height [2]. Adults who were malnourished in early childhood have been found to have impaired intellectual performance, delayed childhood development, reduced capacity for physical work, reduced reproductive capacity and more complicated deliveries in women [3–7]. The first 2 years of life is a critical window of opportunity for prevention of growth faltering and undernutrition through prevention of low birthweight and appropriate infant feeding practices [8].

As the 2005 Innocenti Declaration on infant and young child feeding (IYCF) recognized, appropriate feeding practice during infancy and early childhood is vital for ensuring optimal child health, growth and development [9]. The World Health Organization (WHO) and the United Nations Children’s Fund (UNICEF) set a global strategy for optimal IYCF [10]. The Ministry of Health in Ethiopia has also developed and implemented the IYCF guidelines in 2004 [11]. However, IYCF practices are still suboptimal throughout the globe generally and in Ethiopia in particularly [12–17].

Infant feeding practice is a multidimensional and multi-risk factor practice. Sociodemographic related predictors of infant feeding practices are relatively well researched. The literature consistently show that maternal age, socio-economic status, level of education, marital status and location are associated with infant feeding practices [18–24]. However, research examining psychosocial predictors of IFPs are very limited. The limited studies available showed that psychosocial factors were more predictive of IFPs than demographic factors [25–27].

Among the psychosocial factors, prenatal and postnatal depression consistently predicted components of IFPs such as timely breastfeeding initiation i.e. in the first hour of birth [28, 29], exclusive breastfeeding [30–32], breastfeeding duration [33] complementary feeding timely initiation [27] and dietary diversity [34, 35]. Moreover, researchers reported that other psychosocial factors such as intimate partner violence and maternal social support are important predictors of infant feeding practices. While intimate partner violence negatively influences IFPs [36–38], good perceived social support improved appropriate infant feeding practices [39, 40]. On the other hand, several researchers reported intimate partner violence [41–45] and maternal social support [46–49] as positive and negative predictors of maternal depression respectively.

The limited researches available investigating the association between psychosocial factors and IFPs were based on one or two IFP components at a time rather than using child feeding index. This leads to fragmented information and did not show the effect of psychosocial factors on overall feeding patterns. Child feeding index is a composite indicator which allows to measure IYCF practices in their entirety [50–52]. Reinbott et al. reported that a child feeding index is superior to WHO IYCF indicators in explaining length-for-age Z-scores of young children [53]. However, the use of IYCF indices has gained impetus only after early 2000’s and to the authors’ best knowledge there have been no study that utilized IFP indices to explore the effect of maternal depression on infant feeding practice. Thus, the main objective of this study is to determine the longitudinal association of maternal depressive symptoms and other selected psychosocial factors with IFPs in rural Ethiopia using summary IFP indices.

## Methods

This manuscript is based on ENGINE birth cohort study data. The ENGINE birth cohort study is a prospective, community-based study within Empowering New Generations to Improve Nutrition and Economic opportunities (ENGINE) program. ENGINE was a 5 years nutrition intervention program funded by United States Agency for International Development (USAID) implemented from September 2011 to September 2016 in 100 selected districts in rural Ethiopia. Its main goal was to improve nutritional status of mothers’ and young children through a multi-sectoral approach targeting health, nutrition and agriculture. The program conducted operational researches as part of its rigorous evaluation to generate evidence on program performance and impact. The ENGINE birth cohort study was one of the operational researches conducted under this program. The study was led by Tufts University in partnership with Jimma and Hawassa Universities and Ethiopian Public Health Institute. The study aimed to investigate the benefits of an integrated nutrition program and its co-location with agricultural growth program on household agricultural production and productivity, food security, diet diversity, socio-economic status and livelihoods, as well as health and nutritional status of mothers and their children.

The study had an open cohort design, with recruitment and follow up of pregnant women happened for a period of 2 years. It was conducted from March 2014 to March 2016 in three Districts (Woliso, Tiro-Afeta and Gomma) in the South Western part of Ethiopia. Considering 30% attrition rate, a total of 4680 pregnant women were recruited between 12 and 32 weeks of gestation. The data was collected at the lowest administrative cluster (Kebele) level. A total of 117 clusters with a total sample size of 40 pregnant women per cluster were included in the study. All Kebeles within a district were sampled, with the exception of a few excluded due to inaccessibility, and a total of 1560 pregnant women were recruited in each of the three districts. In each study Kebele, study participants were recruited consecutively until the quota of 40 pregnant women was achieved. Mothers with serious medical conditions, early pregnancy termination, multiple pregnancies, stillbirth and newborns with congenital anomalies were excluded from the study/follow up.

Data was collected once during pregnancy for all women (twice for those in the first trimester), at birth, and then every 3 months until the child was 12 months old. Data collection was conducted by trained nurses electronically using Open Data Kit (ODK) software on handheld tablets and submitted to a secured server via an internet connection.

### Measures

#### Infant feeding practices (IFPs

Infant feeding index was constructed using data collected at birth and then every 3 months until 12 months of age. Mothers were asked about timing of breastfeeding initiation, colostrum feeding, anything given to the infant before giving breast milk, whether the infant was still breastfed, number of times the infant was breastfed during the day and night yesterday, and what the infant ate yesterday. Based on these information, five separate IFP indices were prepared to assess age-specific infant feeding practices; namely, within 3 days of birth, at 3 months, 6 months, 9 months and 12 months of child age.

The indices were computed following the methods suggested by Ruel and Menon [52]. Each item was scored depending on whether a practice was appropriate based on the WHO infant feeding recommendations [17, 54]. A practice that was appropriate for a specific age group received a score of 1, and a practice that was inappropriate received a score of 0. Practices that are considered particularly relevant for a given time point received a score of 2 or 3. For example, breastfeeding received a score of 2 for an infant from birth to 12 months of age. A score of 0 was given to non-breastfed infants. Use of bottle with a nipple was scored as 0 because the practice is considered inappropriate for all age groups; avoidance of infant bottles received a score of 1, indicating an appropriate practice. The dietary diversity score was calculated by adding the number of food groups consumed in the last 24 h and received a score of 0 if the child got below three food groups, 1 if the child got three food groups, and scored 2 if the child got four or more than four food groups in the past 24 h (Table 1).

**Table 1:** Feeding practice variables and scoring system used to construct the infant feeding practice index, Ethiopia, 2019

IYCF indicators	Scoring				
	Birth	3 months	6 months	9 months	12 months
Timely Initiation of breastfeeding	0 = after 1 day				
	1 = within 24 h				
	2 = within an hour				
Prelacteal feeding	0 = Yes				
	1 = No				
Colostrum feeding	0 = No				
	1 = Yes				
Exclusive breastfeeding	0 = No	0 = No			
	2 = Yes	2 = Yes			
Frequency of breastfeeding per day	0 = No breastfeeding	0 = No breastfeeding	0 = No breastfeeding	0 = No breastfeeding	0 = No breastfeeding
	1 = less than 8 times	1 = less than 8 times	1 = less than 8 times	1 = less than 8 times	1 = less than 8 times
	2 = 8–11 times	2 = 8–11 times	2 = 8–11 times	2 = 8–11 times	2 = 8–11 times
	3 = > 12 times	3 = > 12 times	3 = > 12 times	3 = > 12 times	3 = > 12 times
Continued Breastfeeding at 1 year	0 = No	0 = No	0 = No	0 = No	0 = No
	1 = Yes	1 = Yes	1 = Yes	1 = Yes	1 = Yes
Bottle feeding	0 = Yes	0 = Yes	0 = Yes	0 = Yes	0 = Yes
	1 = No	1 = No	1 = No	1 = No	1 = No
Introduction of solid/semi-solid/soft food	0 = Yes	0 = Yes	0 = No	0 = No	0 = No
	1 = No	1 = No	2 = Yes	2 = Yes	2 = Yes
Minimum dietary diversity				0 = less than 3 food types	0 = less than 3 food types
				1 = only 3 food types	1 = only 3 food types
				2 = 4 & more food types	2 = 4 & more food types
Minimum meal frequency				0 = no food taken	0 = no food taken
				1 = 1–2 times a day	1 = 1–2 times a day
				2 = 3 or more times	2 = 3 or more times
Minimum acceptable diet				0 = No	0 = No
				1 = Yes	1 = Yes
Consumption of iron rich food				0 = No	0 = No
				1 = Yes	1 = Yes
On demand BF	0 = No	0 = No	0 = No	0 = No	0 = No
	1 = Yes	1 = Yes	1 = Yes	1 = Yes	1 = Yes
Active feeding			0 = No	0 = No	0 = No
			1 = Yes	1 = Yes	1 = Yes
Potential score	0–13	0–9	0–9	0–15	0–15

The unstandardized total score could reach a maximum of 9–15 scoring points depending on the time point. The indices were standardized by converting each score into percentage of the maximum total score of the scales at each time point. A higher score in the feeding scales indicated a better infant feeding practice. The index was treated as continues variable. Table 1 below depicts the infant feeding practice variables and scoring system used in this paper.

#### Maternal depressive symptoms

Maternal depressive symptoms were assessed using the Patient Health Questionnaire (PHQ-9) [55]; once during pregnancy for all women (twice for those in the first trimester), within 72 h after birth and 3 months postpartum. The PHQ-9 is a 9-item self-administered questionnaire designed to evaluate the presence of depressive symptoms during the prior 2 weeks. The nine items of the PHQ-9 are based directly on the nine diagnostic criteria for major depressive disorder in the DSM-IV (Diagnostic and Statistical Manual Fourth Edition) [56]. Each of the nine items can be scored from 0 (not at all) to 3 (nearly every day). Thus, the total score can range from 0 (absence of depressive symptoms) to 27 (most severe depressive symptoms).

The PHQ-9 scale had been validated for Afaan Oromo Language in a similar population prior to the commencement of the ENGINE birth cohort study and possessed good psychometric properties. A PHQ-9 score of 8 or above was taken as a cut off to define depressive symptoms [57]. For this study maternal depressive symptoms were classified as prenatal, postnatal and persistent. Only few mothers have time point two data and hence only time point one depressive symptoms data were used to define prenatal depressive symptoms. Depressive symptoms measured within 3 days of birth were used to define postnatal depressive symptoms. Whereas, persistent depressive symptoms were defined as mothers screened positive for depressive symptoms during all the three assessment periods; during pregnancy, at birth and 3 months postpartum. Only 1.2% of the participating women had persistent depressive symptoms and this category of depressive symptoms was not considered in the final model.

#### Household food insecurity

The household food insecurity was measured using the Household Food Insecurity Access Scale [58] at baseline (recruitment), at infants 6 months and 12 months of age. For this article we used the baseline measurement. The index women were asked nine questions (yes/no) to determine if anyone in their household had experienced problems of food access over 4 weeks preceding the interview. An affirmative response to any of the nine questions was followed by a question to determine how often the condition happened: rarely (1–2 times), sometimes (3–10 times), and often (> 10 times). Responses were coded as 0 = never (i.e., no experience), 1 = rarely, 2 = sometimes, or 3 = often. Household food insecurity was categorized into four severity levels: food secure, mildly food insecure, moderately food insecure, and severely food insecure as per the algorithm described by Coates et al. [58].

#### Intimate partner violence (IPV)

A screening tool called HITS (Hurt, Insult, Threaten and Scream) was applied to assess intimate partner violence [59]. This data was collected from mothers within 3 days of birth. The scale has four items and each item was scored on a scale of 1 (never) to 5 (frequently) with total score of 20 possible. Then, sum score was computed and treated as a continuous variable in the model.

#### Maternal social support

Maternal Social support was measured using the Maternity Social Support Scale (MSSS) developed by Webster and colleagues [60] within 3 days of birth. The scale contains six items. Each item has measured on a five-point Likert scale of 1 (never) to 5 (frequently) and a total score of 30 was possible. Similarly, the score was treated as continuous variable in this study where a high score corresponds with a high level of perceived social support.

#### Sociodemographic characteristics

Educational status of the mother was categorized into four as illiterate, primary, junior and secondary and above for analysis purpose. Marital status was dichotomized into married (married monogamous and married polygamous) and unmarried (single, widowed, divorced, and separated). Religion was categorized into three as Muslim, Protestant, and Catholic & Orthodox. Similarly, mothers’ age was categorized as < 25 years, 25–35 years and above 35 years. Gestational age at birth was dichotomized as term (37 weeks and above) and preterm (< 37 weeks). Birthweight dichotomized as normal (2500 g and above) and low birthweight (< 2500 g); however, birthweight was treated as a continuous data in the model. A wealth index was created following the methods described by the Demographic and Health Surveys for Ethiopia [21] using polychoric principal component analysis to represent a composite measure of a household’s cumulative living conditions and then separated into quintiles.

### Statistical analysis

We examined whether missing data on feeding practices and maternal depressive symptoms differed from those who were not missing these data. We compared these two groups on infants’ birth weight, household food security, and other key baseline sociodemographic variables. For the continuous variables, we used a t test for equality of means, and, for the categorical variables, we used Pearson’s chi-square tests.

Participants’ characteristics, IFPs and maternal depressive symptoms were summarized using descriptive statistics. To assess longitudinal relationship of infant feeding practice (IFP) and maternal depressive symptoms, we assumed that the repeated measurements of IFPs taken from each infant, overtime, are correlated and it is expected that study participants changed feeding practices over time as infants gets older. To examine differences in IFP within individual subjects over the follow up period, a linear multilevel mixed effects (fixed effects and random effects) model with a random intercept and a random slope was fitted with maximum likelihood estimation method. The fixed effects describe a population intercept and population slopes for a set of covariates, which include exposures and potential confounders. Random effects describe individual variability in IFP and changes over time. By considering individual random slopes and intercepts, this model allows to examine the influence of covariates on the change in IFP over time. Subjects with IFP data from at least two assessment intervals were included in the analysis.

## Results

### Missing data and attrition

Of the total 4680 pregnant women recruited and followed-up, 1090 study participants (23.3%) were lost to follow-up between recruitment and 12 months’ postpartum. The most lost to follow up (47.7% of the total lost to follow) occurred at time point three. The main reasons for the lost to follow-up were stillbirths (3.3%), infant death unrelated to the study (3.0%), twin pregnancy (1.3%), abortion (0.9%) and others such as relocation, refusal to continue participation, and absence during data collection after three repeated trial (6.7%). The data collection was stopped before 378 (8.1%) of the infants reached 12 months of age and hence time point seven data missed for these participants. Potential biases related to missing data were assessed based on infant and maternal demographic and socio-economic characteristics. Study participants with missing data on maternal depression and IFPs did not differ from participants with complete data on basic sociodemographic or other study variables.

### Characteristics of the study participants

Characteristics of the study participants are presented in Table 2 below. The median age of study participants at the time of recruitment was 26 years (inter-quartile range [IQR] 22, 30). More than half of the pregnant women (55.2%) were illiterate and only 241(5.1%) of the respondents had completed secondary education or higher. Just over two-third (67.3%) of the respondents were Muslim and 97.7% were married. The infant girls were slightly higher than boys in number. Over 95% of the infants were normal birthweight (weighing greater than 2.5 kg), however, more than a third of them were born preterm. In terms of morbidity, 57.5% of babies were ill at least once during the infancy period (Table 2).

**Table 2:** Characteristics of study participants, Ethiopia, 2019

Variables	Number	Percent
Maternal age		
Less than 25 years	1615	34.5
25–35 years	2831	60.5
Above 35 years	234	5.0
Median (IQR)	26(22–30)	
Religion		
Orthodox	1057	22.6
Protestant & Catholic	472	10.1
Muslim	3148	67.3
Maternal educational status		
Illiterate	2585	55.2
Primary	1491	31.9
Junior	363	7.8
Secondary & above	241	5.1
Marital status		
Married	4572	97.7
Unmarried	108	2.3
Infants gender		
Male	2252	49.7
Female	2276	50.3
Birthweight		
Normal birth weight	3972	95.6
Low birth weight	183	4.4
Gestational age at birth		
Preterm	1442	34.7
Term	2717	65.3
Child illness during the infancy period		
Yes	2690	57.5
No	1990	42.5
Antenatal care		
No ANC	1626	34.8
1–3 ANC visits	1194	25.5
4 plus visits	1859	39.7
Place of delivery		
Health facility	2934	66.4
TBA	75	1.7
Home	1345	30.4
Other	66	1.5
Birth complication		
Yes	927	19.8
No	3753	80.2
History of spontaneous abortion		
Yes	539	11.5
No	4140	88.5
History of child death		
Yes	1213	25.9
No	3466	74.1
Intimate partner violence (total score > 10)		
Yes	417	8.9
No	4263	91.1
Maternal social support		
Good	2631	56.2
Poor	2049	43.8
Maternal social participation		
Yes	2886	61.7
No	1794	38.3
Household food insecurity		
Secured	1600	34.2
Mildly insecure	600	12.8
Moderately insecure	1846	39.4
Severely insecure	634	13.5
Wealth index		
Lowest	928	19.8
Second	957	20.4
Middle	863	18.4
Fourth	986	21.1
Highest	934	20.0

### Infant feeding practices

Patterns of IFPs are presented in Table 3 below. The findings of the present study demonstrated that in Ethiopia infant feeding practices is generally poor, especially after the first 6 months of age. Dietary diversity and consumption of iron rich foods are particularly unacceptable low. However, majority of mothers follow the WHO recommended breastfeeding practices; namely, early initiation of breastfeeding, colostrum feeding, prelacteal feeding, exclusive breastfeeding and continued breastfeeding at 1 year are relatively better (Table 3).

**Table 3:** Core infant feeding practices, Ethiopia, 2019

Infant feeding practices indicators		Number	Percent
**Core indicators**			
Timely Initiation of breastfeeding	Within an hour	3031	72.5
	Within 24 h	1048	25.1
	After 1 day	100	2.4
Exclusive breastfeeding at:	Within 3 days of birth	4097	97.2
	3 months	2936	89.3
Continued breastfeeding at 1 year		3531	99.6
Introduction of solid/semi-solid/soft food at:	Within 3 days of birth	8	0.2
	3 months	217	5.2
	6 months	1707	42.1
	9 months	3605	90.2
	12 months	3442	95.7
Minimum dietary diversity at:	6 months	13	0.8
	9 months	27	0.7
	12 months	88	2.6
Minimum meal frequency at:	6 months	1405	82.5
	9 months	1914	53.1
	12 months	2158	62.7
Minimum acceptable diet at:	6 months	4	0.4
	9 months	12	0.3
	12 months	16	0.3
Consumption of iron rich food	6 months	4	0.2
	9 months	12	0.3
	12 months	16	0.3
**Optional indictors**			
Frequency of breastfeeding at:			
Within 3 days of birth	< 8 months	1007	24.6
	8-11 months	1880	46.0
	> = 12 months	1200	29.4
3 months	< 8 months	340	8.3
	8-11 months	1802	44.2
	> = 12 months	1935	47.5
6 months	< 8 months	470	11.8
	8-11 months	1753	44.0
	> = 12 months	1766	44.3
9 months	< 8 months	624	15.9
	8-11 months	1907	48.5
	> = 12 months	1397	35.6
12 months	< 8 months	864	24.6
	8-11 months	1669	47.5
	> = 12 months	976	27.8
Colostrum feeding		3653	87.4
Prelacteal feeding		36	0.9
Bottle feeding	Within 3 days of birth	136	3.1
	3 months	635	15.2
	6 months	1394	34.4
	9 months	1406	35.2
	12 months	992	27.6
On demand BF	Within 3 days of birth	3280	78.3
	3 months	2662	64.4
	6 months	2189	54.4
	9 months	1765	44.6
	12 months	1321	37.3
Active feeding	6 months	972	56.9
	9 months	2114	58.6
	12 months	2050	59.6

The study shows that considerable number of infants were exclusively fed only breast milk beyond 6 months of age. Using the 24-h recall methods, 14.6% (*n* = 683) and 11.3% (*n* = 530) of infants at nine and 12 months of age respectively, were still exclusively fed breast milk. However, when plausibility was checked against introduction of solid and semi-solid food and exclusive breastfeeding status during the previous assessments (time points), this prevalence reduced to 1.6% (*n* = 64) and 0.1% (*n* = 3) at nine and 12 months in that order.

### Maternal depressive symptoms prevalence

Among women who screened for depressive symptoms, cumulative incidence proportions were 10.8, 18.5 and 7.5% during pregnancy, within 3 days after birth and 3 months’ postpartum, respectively. Overall, 1156 (26.2%) of the mothers had depressive symptoms at least once during the period between recruitment and 3 months’ postpartum and 56 respondents (1.2%) were having depressive symptoms persistently during the three measurement times. PHQ-9 mean scores (standard deviation) during the three occasions were 3.11 (4.53), 4.35 (4.04) and 2.13 (3.20) respectively.

### Longitudinal relationship of IFP and maternal depressive symptoms

Based on the standardized composite indices of IFP, the poorest IFP practice occurred at 6 months of infancy (only 54% of the potential score). The practice was better during the first 6 months than the second half of the infancy period (Table 4).

**Table 4:** Descriptive statistics of infant feeding practices by infants’ age, Ethiopia, 2019

Time point	Subject	Potential score	Mean, SD (Range)	
			Unstandardized	Standardized
Birth	4023	0–13	10.4 ± 1.1 (5–13)	79.7 ± 8.6 (38.5–100)
3 months	3120	0–9	7.3 ± 1.5 (2–9)	81.5 ± 17.0 (22.2–100)
6 months	3987	0–10	5.4 ± 1.3 (2–10)	53.9 ± 12.9 (20.0–100)
9 months	3543	0–15	8.5 ± 1.3 (5–14)	56.5 ± 8.9 (33.3–93.3)
12 months	3355	0–13	7.4 ± 1.2 (4–12)	56.7 ± 9.6 (30.8–92.3)

As presented in Table 5, linear mixed effects model showed that postnatal maternal depressive symptoms were negatively associated with IFPs (*P* = 0.001). However, prenatal maternal depressive symptoms were not associated with IFPs (*P* = 0.953). Similar to postnatal depressive symptoms, intimate partner violence was negatively associated with IFPs (*P* = 0.001). On the other hand, maternal social support (*P* = 0.002) and social participation (*P* < 0.001) were positively associated with infant feeding practices. Compared with Orthodox Christians, Protestant & Catholic Christians have better IFPs scores (*P* = 0.002) but infants from Muslim families have poorer IFP scores (*P* < 0.001). Contrary to expected, moderate household food insecurity (*P* = 0.003), severe household food insecurity (*P* = 0.01) and infant morbidity episodes (*P* = 0.013) were positively associated with infant feeding practices. Maternal education and gestational age at birth were other important factors positively associated with IFPs in this study.

**Table 5:** Multilevel model results of the association between infant feeding practices and maternal depressive symptoms and other predictors, Ethiopia, 2019

Factors	β	95% CI		*P*-value	SE
Maternal age	0.010	−0.036	0.057	0.66	0.024
Mother Education					
Illiterate					
Primary	0.787	0.240	1.333	**0.005**	0.279
Secondary & above	1.357	0.567	2.146	**0.001**	0.403
Religion					
Orthodox					
Protestant & Catholic	1.497	0.548	2.447	**0.002**	0.484
Muslim	−2.587	−3.209	−1.965	**< 0.001**	0.317
Wealth index	−0.007	−0.073	0.060	0.844	0.034
Household food security					
Secured					
Mildly insecure	−0.692	−1.458	0.073	0.076	0.391
Moderately insecure	0.836	0.293	1.380	**0.003**	0.277
Severely insecure	1.034	0.251	1.816	**0.01**	0.399
Prenatal Depression					
Yes	−0.024	−0.802	0.755	0.953	0.397
No					
Postnatal Depression (within 3 days of birth)					
Yes	−1.031	−1.647	−0.414	**0.001**	0.314
No					
Maternal social support	0.107	0.041	0.174	**0.002**	0.034
Social participation	0.552	0.298	0.806	**< 0.001**	0.129
Intimate partner violence	−0.208	−0.337	−0.080	**0.001**	0.065
Child gender					
Male					
Female	−0.128	0.580	0.326	0.58	0.232
Gestational age at birth	0.517	0.035	0.998	**0.036**	0.246
Antenatal care visits					
No ANC					
1–3 ANC visits	−0.418	−1.023	0.186	0.175	0.309
4 plus visits	0.261	−0.325	0.847	0.382	0.299
Child illness					
Yes	0.625	0.134	1.117	**0.013**	0.251
No					
Constant	67.389	64.723	70.054	< 0.001	1.360
**Random-effects**					
Variance of random intercept	7.208	6.971	7.453		0.123
Variance of random slope	24.743	23.741	25.789		0.522
Covariance of random intercept and slope	−0.998	−0.999	−0.994		0.001
Variance of measurement errors	13.29416	13.09836	13.49288		0.10064

*SE* standard error

## Discussion

In this study we examined the association between maternal depressive symptoms and other psychosocial factors with IFPs using a computed index suggested by Ruel and Menon [52]. The key contribution of this study is to show the effect of maternal depressive symptoms and other psychosocial factors on IFPs in rural Ethiopia. The current findings demonstrated that postnatal depressive symptoms and intimate partner violence predicted poor infant feeding practices. The findings also show that strong maternal social support and active social participation were associated with better infant feeding practices. These findings have important implications for policy makers, researchers, donors and program implementers working on child nutrition in Ethiopia, where the burden of child malnutrition is the highest [14].

A statistically significant negative association was found between postnatal depressive symptoms and overall IFP score in this study. So far, only a few studies have used IFP index in feeding practice studies and to the researchers’ best knowledge there is no study which explores the longitudinal relationship between IFPs and maternal depressive symptoms using an IFP index. However, several previous observational studies reported that maternal postnatal depression is associated with specific components of IFPs, though, the direction of association between breastfeeding and postpartum depression remains unclear [61]. Systematic reviews in 2019 and 2015 concluded that depressed women breastfed their children for shorter duration than non-depressed women [30, 31]. Other previous studies also reported a negative association of maternal depression with early initiation of breastfeeding [28], timely complementary feeding initiation [27] and infants’ dietary diversity [34, 35].

Infants born to women who experienced intimate partner violence were at greater risk of poor infant feeding practices. This finding is consistent with previous studies [36–38] and has important implications, particularly in Ethiopia, where 34% of ever-married women experienced such violence [14]. There are many pathways through which intimate partner violence can affect infant feeding practices [62]. First, IPV is a stressor which leads to depression [42, 63, 64]. As stated above, maternal depression leads to poor infant feeding practices [31]. IPV can also indirectly affect IFPs by influencing mothers’ exposure to antenatal care services [65] and hence access to nutrition related counseling. Studies have suggested that in resource limited settings quality prenatal and delivery care services significantly increase early initiation and duration of exclusive breastfeeding in the first 6 months of life [66]. According to WHO, women who suffer from IPV face social problems, lack of family support, restricted access to services, strained relationships with health care providers and employers, and isolation from social networks [67]. As a result, affected mothers initiate prenatal care later or receive inadequate care or no antenatal care at all [68, 69]. Another pathway could be that mothers with low self-esteem or lack of confidence as a consequence of IPV [70] are also less likely to exercise adequate infant feeding practices [71].

Contrary to many of previous studies, we found that infants in moderately and severely food insecure households rather have better IFP scores. Several previous studies reported that household food insecurity was negatively associated with infant feeding practices [72, 73]. However, the direction of the association does not mean that all infants in food secure households received appropriate and adequate feeding. In Uganda, Pascal et al. found that eight out of 10 infants in food secure households were not receiving the minimum dietary diversity required and reported that household food insecurity explains only 10% of the variance of dietary quality determinants [74]. Conversely, our finding agrees with the studies in Kenya and Tanzania [75, 76]; both studies concluded that infants from food insecure households were less likely to receive cow milk before they reached 6 months. Particularly in Kenya, dairy producing households had a 12-fold increased risk for exclusive breastfeeding interruption by early animal milk introduction compared to those in households without cattle.

Another probable reason for the positive association between food insecurity and IFPs in this study could be ENGINE program vulnerable households focused IYCF interventions. ENGINE end-line impact assessment reported that the program achieved over 10 percentage point increase in infant and child feeding index (ICFI) in 50% of intervention Districts [77]. Studies showed that IYCF focused nutrition education for caregivers improved child dietary diversity and nutrition knowledge of caretakers even in food insecure areas [78, 79]. Moreover, as we indicated earlier, the IFP scores were relatively higher during the first 6 months than the second half of infancy; during the first 6 months, the IFP elements are more amenable to improve by IYCF focused social and behavior change communications costing no or minimal resources for a rural mother.

In this study, only half of women reported that they feel they have good social support during pregnancy (43.8%) and postpartum period (56.2%). We found that maternal social support was positively associated with infant feeding practices. In agreement with our findings, previous studies reported that maternal social support helps mothers to practice appropriate infant and young child feeding [39, 40]. Similarly, our study revealed that infants whose mothers actively participated in social groups have a better IFP scores than those with poor participation. Previous studies consistently reported that social participation is associated with mental and physical health benefits. Seeman and colleagues found that having three or more regular social contacts, as opposed to zero to two such contacts, is associated with lower allostatic load scores [80]. Lower allostatic load mean lower depression [81, 82] and then better IFP scores. In Ethiopia social groups are main platforms to reach mothers with IYCF messages [83].

Gestational age at birth was positively associated with IFP scores. This implies that preterm infants were not receiving good IFPs compared to their full term counterparts. Consistent with our findings, previous studies reported that mothers of preterm infants initiated breastfeeding late and that preterm infants are breastfed for a shorter duration [84–86]. Similarly, observational studies in Italy and the United Kingdom reported early introduction of solid foods with a majority of preterm infants receiving a solid food prior to 4 months of age [87, 88].

A systematic review by Kajali and Vector revealed that restriction or interruption of complementary foods during illness is frequent because of children’s anorexia, poor awareness by caregivers’ about the feeding needs of sick children, traditional beliefs and behaviors, and/or suboptimal counselling and support by health workers [89]. However, we found that infants with higher morbidity episodes have higher IFP scores too. We presumed that frequent episodes of illness increase mothers’ frequency of contact with health care providers and hence repetitive IYCF counseling which improves mothers’ IYCF awareness and practices. Abegaze and colleagues reported that in Ethiopia mothers with prior experience of infant illness were more likely to seek healthcare for their sick children than their counterparts [90]. Moreover, as a sick infant loses its appetite, mothers could frequently serve different type of foods to the infant that potentially increases diet diversity and/or frequency and increase the IFP scores.

Mothers with primary and above school qualification seemed to perform better with respect to IFPs than illiterate mothers. This finding is in agreement with previous studies in Ethiopia and elsewhere [13, 28, 34, 35, 91]. This may be explained by educated mothers having better understanding of IYCF itself and/or had exposure to IYCF awareness raising campaigns (through their ability to read leaflets, posters and banners), that have been conducted for several years by the Ministry of Health and development partners in Ethiopia.

The IFPs score is relatively higher during the first 6 months than the second 6 months of age. During the first 6 months, IFP components need no significant additional costs for rural Ethiopian women but mothers’ commitment and knowledge. However, during the second 6 months of the infancy period, the IFP components need resources particularly to fulfill the required quality of meal and the frequency. As revealed in this study, less than 3 % of infants received a quality diet as measured by dietary diversity. Several previous studies in Ethiopia came up with similar findings of unacceptably low percentage of infant dietary diversity [22, 92–94]. Moreover, we found that the IFP score was the poorest particularly at 6 months of age. This might be explained by the small proportion of mothers who practiced timely initiation of complementary feeding in this study. Besides, 6–8 months of infancy is the transition period where mothers/caretakers teach babies to take solid and semi-solid foods.

In this study, using 24-h recall methods, more than 10 % of infants were exclusively feed only breast milk beyond 6 months of age. However, this proportion has substantially dropped when plausibility was checked against introduction of solid/semi-solid/soft food or liquid and exclusive breastfeeding status during the previous assessments (time points). Previous researchers have commented on the drawbacks of the 24-h recall method that it misclassifies too many mothers as exclusively breastfeeding and suggested several techniques that needs to be taken during data collection to minimize the problem [95–98]. Based on our findings, for longitudinal studies, plausibility check against introduction of solid/semi-solid or liquid other than breast milk during previous time points could be used as one option to reduce the downside of point-in-time or current status exclusive breastfeeding measurements (24-h recall).

One of the main strengths of this study is that it is based on community based longitudinal data (prospective birth cohort) with appropriate analytical techniques applied. The study had a large sample size, high response rate and low attrition. Data were collected on regular intervals on several important sociodemographic, nutritional and clinical risk factors that could be harvested for this analysis. In addition, we used 14 WHO recommended IYCF core and optional indicators to compute the IFP score [54]. One limitation of this study is that IFP data were based on mothers/caretakers reports and, thus, are subject to possible recall biases. Moreover, the presence of depressive symptoms may cause mothers to have more negative views about things around them, including household food security, child health and feeding practices.

## Conclusions

The findings of the present study demonstrate that in Ethiopia, infant feeding practices are generally poor, particularly, dietary diversity and consumption of iron rich foods are unacceptable low and two third of the households are food insecure. Moreover, prenatal and postnatal maternal depressive symptoms are quite common. Mainly, the study showed the longitudinal relationship of maternal depressive symptoms and other psychosocial factors with infant feeding practices, whereas postnatal depressive symptoms and IPV negatively associated with IFPs, perceived maternal social support and active social participation positively predicted infant feeding practices.

The implications of our findings for practice are to emphasize the need for prevention, early detection and treatment of postpartum depression, intimate partner violence and to strengthen household food security interventions, so that mothers are healthy, food secured and practicing appropriate infant feeding as per WHO infant feeding recommendation. Depression during pregnancy and postpartum often goes unrecognized by healthcare providers as changes in sleep, appetite and fatigue may be attributed to normal pregnancy and postpartum changes [99]. Furthermore, women often are reluctant to report changes related to depression, for example, Whitton and colleagues reported that over 80% of women diagnosed with postpartum depression had not reported symptoms to healthcare providers [100]. Similarly, IPV victims often experience feelings of shame and isolation and do not communicate openly to others that violence has occurred to them by their spouses [101].

Consequently, during pregnancy and postpartum period, women may particularly benefit from early screening for depression and intimate partner violence. However, screening without subsequent appropriate intervention may be counterproductive as women may find repeated screening difficult and potentially decrease their utilization of other health services. On the other hand, contrary to the expected result, the current study has shown that mothers in food insecure households may implement better infant feeding practices than those in food secure households. Based on this finding, advocating for policies to invest in and promote household food security solely as a means to improve infant feeding practices may have minimal impact without additional social and behavior change components.

In this regard, we recommend the Ethiopian Ministry of Health to integrate postnatal maternal depression and intimate partner violence screening into the routine postnatal care service and for the Ministry of Agriculture together with other relevant ministries, donors and implementers, to strengthen household food security interventions. Moreover, we recommend all relevant stakeholders particularly Ministry of Women Affaires to promote maternal social support and social participation. Overall, we concluded that a multitude of factors are related to IFPs and need coordinated, multi-sectoral and multi-stakeholder interventions.

## Data Availability

The data that support the findings of this study are available from Tufts and Jimma Universities but restrictions apply to the availability of these data, which were used under license for the current study, and so are not publicly available. Data are however available from the authors upon reasonable request and with permission of Tufts and Jimma Universities.

## Abbreviations

CI: Confidence interval
HITS: Hurt, Insult, Threaten and Scream
IFP: Infant feeding practice
IPV: Intimate partner violence
IYCF: Infant and young child feeding
MSS: Maternal social support
OR: Odds ratio
PHQ-9: Patient health questionnaire-9
USAID: United States Agency for International Development
